# Comparison of two alar cinch base suture in orthognathic surgery: a randomized clinical trial

**DOI:** 10.1590/0103-6440202204653

**Published:** 2022-04-29

**Authors:** Thames Bruno Barbosa Cavalcanti, Carolina Chaves Gama Aires, Rosa Rayanne Lins de Souza, Luiz Alcino Monteiro Gueiros, Ricardo José de Holanda Vasconcellos, Jair Carneiro Leão

**Affiliations:** 1 Department of Clinical and Preventive Dentistry, Federal University of Pernambuco, Pernambuco, Brazil.; 2 Department of oral and maxillofacial surgery, Dental School of Pernambuco. University of Pernambuco (FOP/UPE). Recife, Pernambuco, Brazil

**Keywords:** Orthognathic Surgery, alar base, alar base cinch

## Abstract

Le Fort I osteotomy is widely used in orthognathic surgery to correct maxillary deformities. However, this osteotomy may be related with the increase of alar base width. The aims of the present study were to compare two alar cinch suture after Le Fort I osteotomy and observe which type presents a better result in controlling the enlargement of the alar base after maxillary repositioning in orthognathic surgery. A randomized clinical trial was carried out with 40 patients randomly assigned in two intervention groups: group 1 - patients submitted to internal suture and group 2 - patients submitted to external suture. Of the 40 patients, 65% were female and 35% were male. The mean age of the patients was 30,25 in group I and 28,6 in group II. There was an increase in the alar base width in both groups, with significant difference between the means (P < 0,001). It was possible to compare the evolution of the means of the alar base width between group I and group II. Thus, it was observed that the external technique (group II) better controlled alar base width after Le Fort I osteotomy. It was not possible to relate the enlargement of the alar cinch with maxillary movement performed (P > 0,05). Overall, alar base cinch suture is an essential component of Le Fort osteotomies to control the alar base width. In this study, the external technique was more effective when compared to the internal technique in controlling the enlargement of the alar base width.

## Resumo

A osteotomia Le Fort I é amplamente utilizada em cirurgia ortognática para corrigir as deformidades maxilares. No entanto, esse tipo de osteotomia pode estar relacionado ao aumento da largura da base alar. Os objetivos do presente estudo foram comparar duas técnicas de sutura da base alar após a realização da osteotomia Le Fort I, bem como observar qual tipo apresenta melhor resultado no controle do alargamento da base alar após o reposicionamento maxilar em cirurgia ortognática. Foi realizado um ensaio clínico randomizado com 40 pacientes alocados aleatoriamente em dois grupos de intervenção: grupo 1 - pacientes submetidos à técnica de sutura interna e grupo 2 - pacientes submetidos à técnica de externa. Dos 40 pacientes, 65% eram do sexo feminino e 35% do masculino. A média de idade dos pacientes foi de 30,25 no grupo I e 28,6 no grupo II. Houve aumento da largura da base alar em ambos os grupos, com diferença significativa entre as médias (P <0,001). Foi possível comparar a evolução das médias da largura da base alar entre o grupo I e o grupo II. Assim, observou-se que a técnica externa (grupo II) controlou melhor a largura da base alar após a osteotomia Le Fort I. Não foi possível relacionar o alargamento da base alar com o tipo de movimentação maxilar realizado (P> 0,05). No geral, a sutura da base alar é um componente essencial das osteotomias Le Fort para controlar o alargamento da base alar. Nesse estudo, a técnica externa foi mais eficaz quando comparada à técnica interna no controle do alargamento da largura da base alar.

## Introduction

Correction of face skeletal deformities through the orthodontic-surgical treatment become a safe and predictable option, mainly due to great advances in surgical techniques, such as the use of stable internal fixation and virtual surgery planning, as well as the precise orthodontic treatment in the occlusions preparation ^1^. Thus, this type of treatment has been widely used, where bone movements are millimetrically calculated and performed surgically with excellent three-dimensional results already described in the literature ^2^ ^,^ ^3^ ^,^ ^4^.

Le Fort I osteotomy is the most widely used surgical technique to correct maxillary deformities. This kind of osteotomy allows a new positioning of the jaw bone is obtained after moving it in the three spatial planes ^3^ ^,^ ^5^. This technique involves the section of the maxillary bone in canine, zygomatic and pterygoid pillars, separating it from the remaining fixed part of the face, allowing the exact movement that the maxilla will perform ^2^ ^,^ ^6^. The predictability of bone changes is already well established. Despite the good skeletal results achieved, the effects that soft tissues suffer from orthognathic surgeries are less predictable ^7^. The need to quantify changes in the facial soft tissue and predict the surgical results aims to create a prediction of the interrelationship between the changes in facial soft and bone tissues ^8^. Nasal region is one of the most susceptible areas to changes, being decisive for a harmonious surgical planning ^9^.

The amount and direction of maxillary movement in orthognathic surgeries and their relationship with soft tissues are still not well described in the literature. The muscle strain that occurs during access to Le Fort I osteotomy is a consequence with an unpredictable prognosis, especially in critical situations, such as major advances as maxillary advancement and superior reposition ^10^. Patients who already have the alar base at the limit or above the aesthetic standard can evolve in the postoperative period with excessive enlargement ^11^.

A transoral vestibular approach, to perform the Le Fort I osteotomy, it causes muscular detachment of the nasal region promoting the widening of the alar base ^12^. This enlargement promotes unsightly changes that must be corrected with the plication of the alar base, to return its normal length. Two suture techniques are widely used to reconstitute the alar cinch, one external and one internal ^13^ ^,^ ^14^. The classic technique, also called internal, was first described in 1980 by Millard ^15^ and it is widely used to correct nasal defects in patients with cleft lip. However, it was not until 1982 that Collins and Epker ^16^ described its use in patients undergoing le fort I osteotomy. On the other hand, in 2002, as an alternative to contain the alar enlargement, Shams and Motamedi ^17^ described the external technique.

With the purpose of making this procedure more predictable and reliable, the external technique allows the apprehension of the cutaneous portion laterally the wing of the nose and not only of the subcutaneous fibrocartilage as described in the internal technique ^17^ ^,^ ^18^.

Despite the main indication of orthognathic surgery is a functional improvement, aesthetic component is extremely important and undergoes changes, especially in the alar cinch ^19^. Although there are many publications on soft tissue changes after orthognathic surgery, further prospective studies are needed to stratify confounding factors, such as the amount of movement, age, gender, race, quantity and quality of soft tissues ^20^. In this context, the aims of the present study were to evaluate, through a randomized clinical trial, the enlargement of the nasal base of patients undergoing Le Fort I osteotomy, as well as to compare two techniques of alar cinch suture, after movements performed in bone tissues in orthognathic surgery.

## Material and methods

### Study design and sample

This study consisted in a randomized clinical trial, conducted using the Consolidated Standards of Reporting Trials (CONSORT) guidelines ^21^. The Research Ethics Committee of the Health Sciences Center of the Federal University of Pernambuco (CAAE 81647317.9.0000.5208/ N° 2532236) approved this study. All patients were informed about the content of the research and signed a free informed consent form. The patients in this study referred for treatment of dento-skeletal deformity and came from the oral and maxillofacial surgery department at the Hospital da Restauração, a public hospital in the city of Recife, state of Pernambuco, Brazil.

Patients were selected from March 2017 to March 2019. Inclusion criteria were patients undergo orthognathic surgery of the maxilla with transoral vestibular approach, Le Fort I osteotomy and that in the intraoperative period they would be submitted to alar cinch suture. Exclusion criteria were presence of cleft lip and / or palate, history of facial fracture, or patients undergo rhinoplasty surgery after orthognathic surgery and before final clinical evaluation, postoperative dehiscence from access and participants who withdraw from the survey.

### Sample size

The sample size was defined through a sample calculation, where Gpower 3.1.9.2 software was used to test hypotheses for the difference of means between two groups, assuming an effect size of 1 as a function of the mean of 2.5 mm and 1,26 mm of Ritto's study ^2^. The following parameters were used: 95% confidence interval, 80% test power and 5% error. Considering possible losses, a design effect of 20% was added, determining N = 40. Minimum sample required was 20 patients in each group, totaling a final sample of 40 patients, as there was no loss of sample follow-up.

### Randomization and blinding

All the 40 patients were randomly allocated through lottery of sealed and numbered brown envelopes contained the type of intervention that would be performed, in order to ensure an equal distribution of the participants into two intervention groups: group 1 - patients submitted to internal suture and group 2 - patients submitted to external suture. The envelope was delivered by the evaluator to the surgical team. Since the evaluator was responsible for the pre and post measurements - operative, he remained outside the operating room to ensure blinding. Thus, the evaluator was not aware of the technique that was used in each patient.

### Data collect

**Figure 1:**
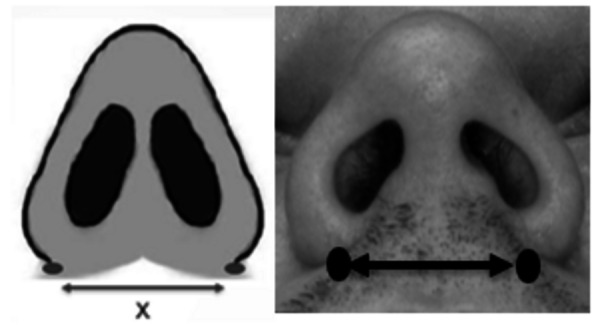
Alar base width reference

A preoperative analysis was performed with a digital pachymeter, where it was measured to the lateral-lateral dimensions of the two craniometric points represented in the alar base (Figure 1) and annotated in the initial care record. The same measurement was performed intraoperatively, in both techniques, at the beginning and at the end of surgery, allowing a faithful statistical analysis between the two groups. After 3 months of postoperative follow-up, a new measure of the alar base was taken for final comparison of the groups.

The same team of surgeons performed all surgeries and were calibrated by the researcher, for standardization of the intraoperative measurements and accurate application of the surgical techniques.

### Protocol of Interventions

All the patients underwent a standard Le Fort I osteotomy under general anesthesia. After nasotracheal intubation, the measures and records of the alar base width was noted by the surgeons. To perform an osteotomy le fort I and posterior repositioning of the maxilla, an incision in the maxillary vestibule is necessary. Transoral vestibular approach involves the detachment of the periosteum superiorly from the pyriform rhyme and the complete mobilization of the alar cinch. After maxillary repositioning and proper osteosynthesis, the alar cinch suture technique was performed, according to randomization. The width of the alar base after the alar cinch suture must coincide with that measurement immediately after nasotracheal intubation. Although the preoperative measurement is essential in the final comparison after 3 months of surgery, intraoperative measurements are also very important, since the presence of the tube changes the diameter of the nostril and can result in erroneous measurements.

In internal technique, fibrocartilaginous tissue was identified by extra-oral pressure on the alar base, followed by bilateral intraoral clamping of this tissue and a suture with a non-absorbable and monofilament Prolene 3-0 suture. During this phase, it was important to observe whether the movement in the wings of the nose was symmetrical. Then, the internal suture was tightened so that the width of the alar base, predetermined after nasotracheal intubation, was reached.

In external technique, a non-absorbable and monofilament Prolene 3-0 suture with a thick needle was used to transect the tissues of the alar base, entering through transoral approach and leaving the skin at the union of the wing of the nose and the upper lip, bilaterally. The needle was then reinserted into the oral cavity through the same exit point on the skin. After crossing the skin, the direction of the needle was changed, without leaving the intraoral incision, and keeping the suture that holds the wing of the nose under the skin. After ensuring the release of the lip and the symmetrical movement in the wings of the nose, alar cinch suture was carefully tightened so that the width of the alar base, predetermined after nasotracheal intubation, was reached.

### Statistical analysis

The data were submitted to a comparative statistical study between groups and different measurement periods. Normality was verified by the Shapiro Wilk’s test. Student's t-tests were applied for independent samples. Pearson correlation coefficient was calculated between the enlargement of alar cinch and the movements performed in the maxilla after Le fort I osteotomy. To verify the significance between motivating factor and the type of alar cinch suture technique used, likelihood ratio test was performed. The statistical software IBM SPSS (Statistical Package for Social Sciences), version 21.0, was used to obtain the results. The significance level was considered when p ≤ 0.05.

## Results

**Figure 2:**
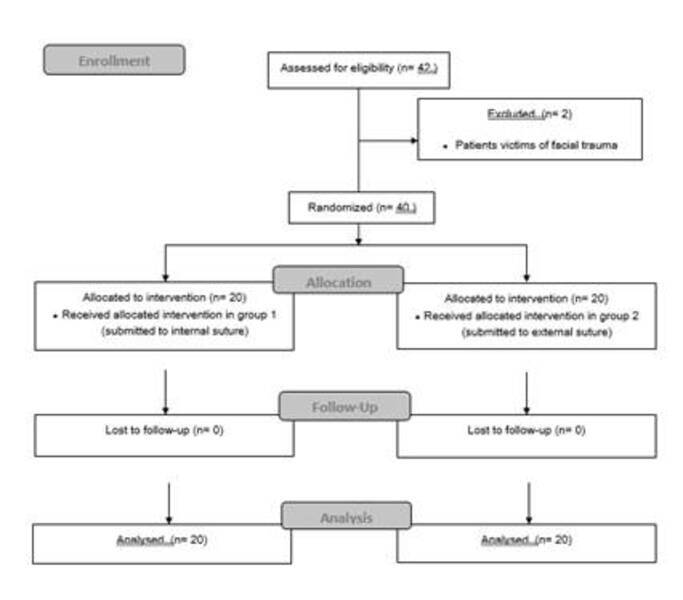
CONSORT Flowchart of inclusion of participants

Forty patients were eligible for this study and were divided into two groups, as shown in the CONSORT flowchart (Figure 2). Of the 40 patients, 26 (65%) were female and 14 (35%) were male. The mean age of the patients was 30.25 in group I (range 18 - 49) and 28,6 in group II (range 18 - 41). Regarding diagnosis of dentofacial deformities 31 patients were class III corresponding to 77.5% of the sample. Concerning motivating factor to undergo orthognathic surgery, 45 % of individuals reported aesthetics as main motivating factor, 27.5% referred function, 25% referred both aesthetics and function complains. Only 1 patient (2.5%) underwent jaw surgery for treat sleep apnea. Likelihood ratio test (Table 1) was performed to verify the significance between the purpose of orthognathic surgery and the type of alar cinch suture technique used but the differences were not statistically significant (p = 0.259).

**Figure 3:**
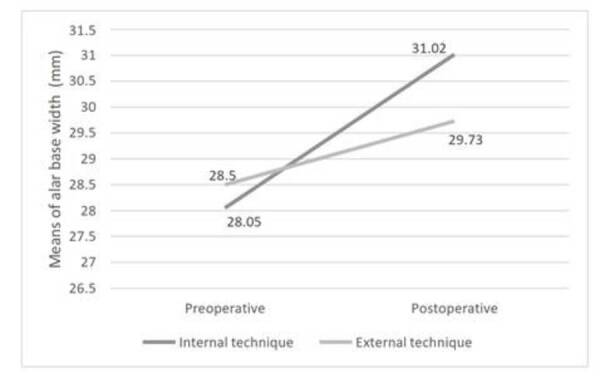
Comparison between preoperative and postoperative means of alar base width between groups

Means of alar base width in preoperative period were similar in both groups, with non-significant difference between the means (P > 0,05) (Table 2). In the postoperative measures, there was an increase in the alar base width in both groups, with significant difference between the means (P < 0.001) (Table 3). It is possible to compare the evolution of the averages of the alar base width between group I and group II. Thus, in the present study, it was observed that the external technique (group II) better controlled alar base width after Le Fort I osteotomy (Figure 3).

**Table 1:** Likelihood ratio test between the purpose of orthognathic surgery and the type of alar cinch suture

Purpose of orthognathic surgery	Technique				Total		p-valor^1^
	Internal		External				
	n	%	N	%	n	%	
Aesthetic	11	55	7	35	18	45	0.259
Function	5	25	6	30	11	27.5	
Aesthetic + function	3	15	7	35	10	25	
Sleep apnea	1	5	0	0	1	2.5	
Total	20	100	20	100	40	100	

**Table 2:** Preoperative measures of alar base width in both groups

	N	Mean	Standard deviation	Minimum	Maximum	p-valor^1^
Internal	20	28.05	3.65	21.74	35.80	0.673
External	20	28.50	3.03	22.48	34.32	

**Table 3:** Differences of alar base width enlargement between groups

	N	Mean	Standard deviation	Minimum	Maximum	p-valor^1^
Internal	20	2.97	0.76	1.50	4.23	<0.001*
External	20	1.23	0.72	0.06	2.87	

Pearson correlation coefficient was used to evaluate a relation between the increase of the alar base width and the movements performed to reposition the maxilla (Table 4). The mean of maxillary advancement was 3,95 mm (range 0 - 10.5); the mean of superior repositioning of the maxilla was 1,52 mm (range 0 - 6); the mean of inferior repositioning of the maxilla was 0.4 mm (range 0 - 2). However, it was not possible to relate the enlargement of the alar cinch with the movement performed (P > 0.05).

**Table 4:** Correlation between enlargement of alar base width and maxillary movements performed

Alar cinch suture	Enlargement	Maxillary movements		
		Advancement	Superior repositioning	Inferior repositioning
Internal	Pearson correlation coefficient	0.101	-0.233	-0.259
	p-valor	0.671	0.324	0.271
	N	20	20	20
External	Pearson correlation coefficient	0.121	-0.196	0.226
	p-valor	0.612	0.408	0.338
	N	20	20	20

## Discussion

Many studies have shown that Le Fort I osteotomy results in nasolabial changes, including enlargement of the alar base width and thinning of the upper lip, and these alterations may be anti-esthetic ^22^. The surgical technique of Le Fort I osteotomy includes procedures performed on bone and cartilaginous parts and soft tissues of the nose that can cause changes in nasal shape and function, and which may sometimes be unpredictable ^23^. In the international literature, there is no consensus as to the best technique for the control of the enlargement of the alar base after orthognathic surgery involving the Le Fort I osteotomy procedure. Therefore, this work sought through the analysis of forty patients submitted to orthognathic surgery with involvement of the maxilla, to identify if the sutures of the nasal base, either by internal or external technique, are necessary and effective.

The suture of the alar base can be used to reduce nasal width increase, and several authors have reported its efficiency and suggest that this procedure limits the widening of the alar base width ^13^. Many modifications of the technique have been described, which makes it difficult to standardize clinical trials and interpret their results, especially with regard to which alar cinch suture is most effective. Two techniques described by Milard ^15^ ^)^ and Shams-Motamedi ^(^ ^17^ are widely used and were compared in the present study. Wolford et al. ^24^ argue that it is easier to correct or control the alar base width at the time of orthognathic surgery rather than submit the patient to another surgical procedure to correct nasal imperfections. However, it is important highlight that alar cinching will not decrease the preexisting alar width. Patients who already have an excess of alar base width in the preoperative period may require a secondary surgical procedure to correct nasal imperfections ^25^.

Some authors consider that soft tissue changes associated with maxillary surgery may be more affected by the position of the soft tissue incision and methods used in closure than by the surgically induced hard tissue change ^26^. However, a certain increase of the alar base width is expected after the Le Fort I osteotomy. A clinical trial performed in 21 patients who underwent orthognathic surgery evaluated the surgical movements and modifications in the nasal tip and alar base. The results showed changes of nasal tip up in 85% of cases, to anterior in 80%, rotation in 80% and increase in the alar base width in 95% of cases ^17^. These results corroborate our findings, since there was an increase in the alar base width in both groups. These findings highlight that the alar base cinch suture is essential in controlling the alar base width after Le Fort I osteotomy and maxillary repositioning ^27^.

The two techniques used in this study to compare the increase of the alar base width were evaluated by others authors. Ritto et al. ^2^ evaluated the modified alar cinch suture, the external technique, and compared with the classic method of alar cinching, the internal technique. This study demonstrated, with a statistically significant difference, that the external technique controlled more effectively the increase of the alar base width. A systematic review also demonstrated the efficacy of a modified alar base cinch suture in maintaining preoperative alar and alar base width ^14^. These studies corroborate the findings of our study, since it was also observed that the external technique better controlled alar base width after Le Fort I osteotomy.

Many authors tried to relate the increase of the alar base width with various maxillary movements. Usually, Le Fort I osteotomies results in widening of the alar bases. Westermark et al. ^28^ analyzed 55 patients who underwent Le Fort I osteotomy for advancement or superior repositioning and found a positive correlation between the alar base width and the degree of maxillary advancement and/or impaction. However, some authors claim that soft tissue change may be caused by the type of the approach to the maxilla and may not be caused by movements of the bone changes that occurs at surgery ^2^ ^,^ ^27^. Unfortunately, we were unable to establish a relation between the increase of the alar base width and the maxillary advancement or superior/inferior repositioning.

Despite relevance of the subject, some limitations such as lack of data about race/ethnicity and a short follow-up can be observed in this study. Although there is no consensus on the follow-up period needed to evaluate the effects post-operative on soft tissues, it has been suggested that this time is variable depending on the region to be evaluated and the alar base width post-operative may be obtained with at least 3 months. However, after 3 months edema may still persist and mask undesirable changes ^29^.

Overall, after Le Fort I osteotomy an increase in the alar base width is expected. Thus, alar base cinch suture is an essential component of Le Fort osteotomies to control the alar base width. The external technique to was more effective when compared to the internal technique for controlling the enlargement of the alar base. Future studies with high methodologic quality, more data from the participants and bone movements performed are necessary.
